# Inhibitory Spike-Timing-Dependent Plasticity Can Account for Pathological Strengthening of Pallido-Subthalamic Synapses in Parkinson’s Disease

**DOI:** 10.3389/fphys.2022.915626

**Published:** 2022-05-19

**Authors:** Mojtaba Madadi Asl, Atefeh Asadi, Jamil Enayati, Alireza Valizadeh

**Affiliations:** ^1^ Department of Physics, Institute for Advanced Studies in Basic Sciences (IASBS), Zanjan, Iran; ^2^ Physics Department, College of Education, University of Garmian, Kalar, Iraq

**Keywords:** inhibitory spike-timing-dependent plasticity, synaptic plasticity, parkinson’s disease, synchronization, basal ganglia

## Abstract

Parkinson’s disease (PD) is a neurodegenerative brain disorder associated with dysfunction of the basal ganglia (BG) circuitry. Dopamine (DA) depletion in experimental PD models leads to the pathological strengthening of pallido-subthalamic synaptic connections, contributing to the emergence of abnormally synchronized neuronal activity in the external segment of the globus pallidus (GPe) and subthalamic nucleus (STN). Augmented GPe-STN transmission following loss of DA was attributed to heterosynaptic plasticity mechanisms induced by cortico-subthalamic inputs. However, synaptic plasticity may play a role in this process. Here, by employing computational modeling we show that assuming inhibitory spike-timing-dependent plasticity (iSTDP) at pallido-subthalamic synapses can account for pathological strengthening of pallido-subthalamic synapses in PD by further promoting correlated neuronal activity in the GPe-STN network. In addition, we show that GPe-STN transmission delays can shape bistable activity-connectivity states due to iSTDP, characterized by strong connectivity and strong synchronized activity (pathological states) as opposed to weak connectivity and desynchronized activity (physiological states). Our results may shed light on how abnormal reshaping of GPe-STN connectivity by synaptic plasticity during parkinsonism is related to the PD pathophysiology and contribute to the development of therapeutic brain stimulation techniques targeting plasticity-induced rewiring of network connectivity.

## Introduction

Parkinson’s disease (PD) is a movement-related disorder that is associated with widespread multi-systemic neurodegeneration (McGregor and Nelson, 2019). Some PD symptoms are related to neuronal loss, whereas others are associated with abnormal neuronal activity (McGregor and Nelson, 2019). Particularly, motor symptoms of PD are linked to significant degeneration of dopaminergic (DAergic) neurons in the substantia nigra pars compacta (SNc) (Brown et al., 2001; Levy et al., 2002; Mallet et al., 2008b). Motor impairment in PD is accompanied by the emergence of excessive neuronal synchronization and abnormal beta band (15–30 Hz) oscillations in the external segment of globus pallidus (GPe) and subthalamic nucleus (STN) (Kühn et al., 2006; Hammond et al., 2007; Mallet et al., 2008a; Neumann et al., 2017; Asadi et al., 2022), that together may play the role of a pacemaker in the basal ganglia (BG) circuitry (Plenz and Kital, 1999; Bevan et al., 2002; Holgado et al., 2010). Abnormal rhythmogenesis in the recurrently connected GPe-STN network occurs following the cascade of structural and functional changes after dopamine (DA) loss (Day et al., 2006; Moran et al., 2011; Fan et al., 2012; Miguelez et al., 2012; Lemos et al., 2016; Chu et al., 2017; Madadi Asl et al., 2022). This ultimately leads to the dysfunction of cortico-BG-thalamo-cortical (CBGTC) loop, as implicated in several movement disorders (DeLong and Wichmann, 2007).

**FIGURE 1:**
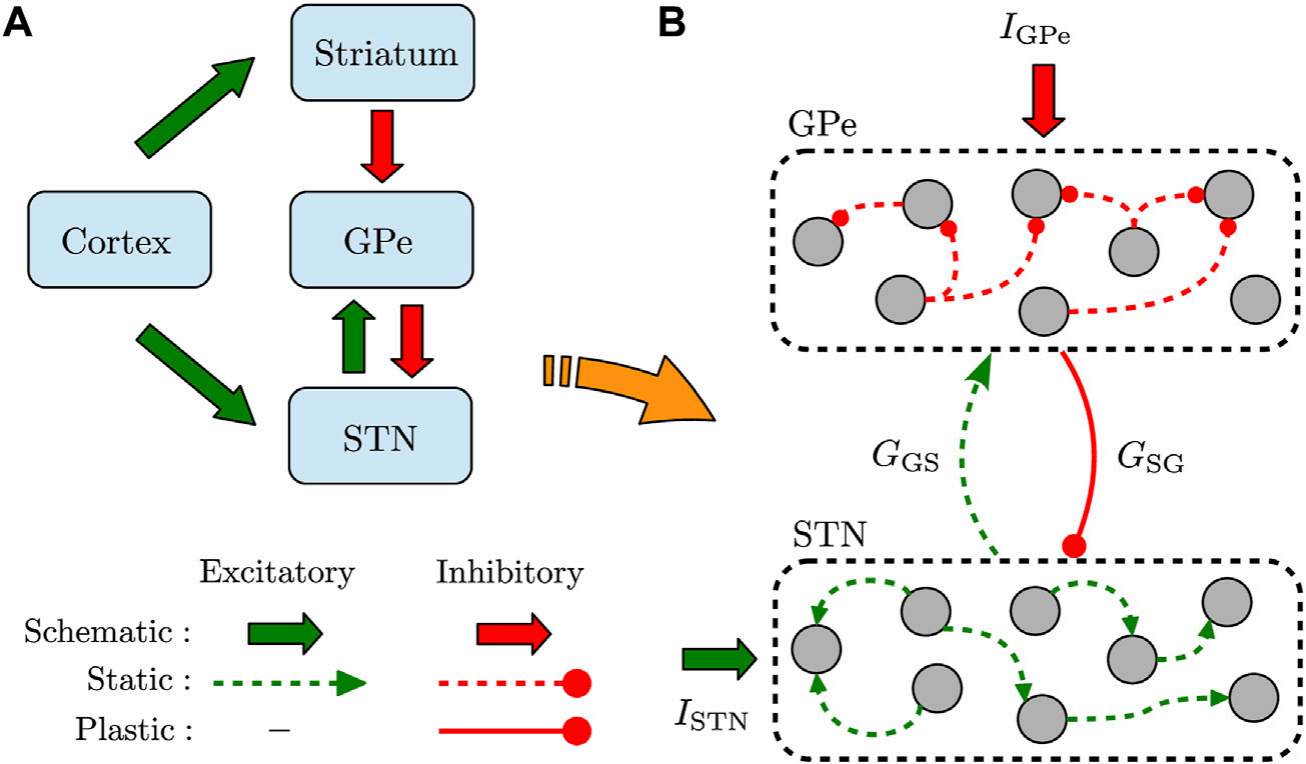
Schematic illustration of the recurrently connected GPe-STN network. **(A)** GPe receives inhibitory input from striatum via the indirect pathway and relays it toward STN. Furthermore, STN receives excitatory input from cortex via the hyperdirect pathway. **(B)** Structure of the GPe-STN network where only the synaptic connections in the pallido-subthalamic pathway (solid red) are modified according to iSTDP. *G*_SG_ (*G*_GS_) denotes the mean GPe → STN (STN → GPe) synaptic strength, and *I*_GPe_ (*I*_STN_) represents the external current applied to GPe (STN).

It is widely accepted that the GPe-STN network within the BG is mediated by cortical inputs via two pathways (see Figure 1A) (McGregor and Nelson, 2019): In the indirect pathway, striatum receives cortical excitation and sends inhibitory inputs to STN through GPe (i.e., cortex → striatum → GPe → STN), whereas the cortical excitation is directly conveyed to STN via the hyperdirect pathway (i.e., cortex → STN). The output of STN is then transmitted towards the BG output nuclei and, consequently, to the thalamo-cortical circuits. The findings of experimental PD models revealed that synaptic transmission in the indirect pathway (Fan et al., 2012; Miguelez et al., 2012; Lemos et al., 2016) and hyperdirect pathway (Chu et al., 2017, 2015) is altered following DA loss. This can result in the imbalance of excitation and inhibition converging towards STN, leading to abnormally synchronized neuronal activity in the GPe-STN network (Galvan et al., 2015). This excessive neuronal synchronization is then propagated to the BG output nuclei, contributing to the motor dysfunction in PD (Hammond et al., 2007; Galvan and Wichmann, 2008).

In particular, Fan and colleagues (Fan et al., 2012) found that the strength of pallido-subthalamic (GPe-STN) synapses are significantly increased following the degeneration of midbrain DAergic neurons in 6-hydroxydopamine (6-OHDA)-lesioned rodents. This proliferation occurred due to an increase in both the number of synaptic connections per GPe-STN axon terminal and their strengths in lesioned rodents compared to control condition (Fan et al., 2012). It has been hypothesized that the STN balances cortical excitation (in the hyperdirect pathway) and pallidal inhibition (in the indirect pathway) likely by an intrinsic homeostatic mechanism (Chu et al., 2015). Excessive engagement of this regulatory mechanism during parkinsonism leads to the pathological strengthening of pallido-subthalamic synapses by heterosynaptic long-term potentiation (hLTP), as demonstrated in 6-OHDA-lesioned rodents (Chu et al., 2015, 2017).

However, synaptic plasticity may play a role in the pathological strengthening of GPe-STN synapses following DA depletion; for a comprehensive review see Madadi Asl et al. (2022). First, altered striato-pallidal (Day et al., 2006; Lemos et al., 2016) and pallido-pallidal (Miguelez et al., 2012) synaptic transmission can reduce GPe-STN activity (Magill et al., 2001; Mallet et al., 2012), resulting in an elevated activation of STN N-methyl-D-aspartate receptors (NMDARs) which further promotes the strengthening of GPe-STN inputs (Chu et al., 2017, 2015). Second, abnormally correlated activity of GPe neurons and STN neurons in PD state (Mallet et al., 2008a) suggests that NMDAR-dependent synaptic plasticity may play the role of classical coincidence detectors in this process where parkinsonian GPe- STN activity would both promote and be promoted by this form of plasticity (Chu et al., 2017, 2015). In fact, experimental observations in parkinsonian rodents showed that the knockdown of STN NMDARs prevents the strengthening of GPe-STN synapses (Chu et al., 2015). Therefore, we hypothesized that spike-timing-dependent plasticity (STDP) at GPe-STN synapses can explain the pathological strengthening of GPe-STN synapses during parkinsonism. Such adaptive changes in synaptic connectivity can promote abnormal neuronal activity in the GPe-STN network.

It is shown that STDP can lead to the emergence of bistable dynamical states due to the coevolution of activity-connectivity patterns (Aoki and Aoyagi, 2009; Gilson et al., 2009; Madadi Asl et al., 2018a) which computationally relate to normal and parkinsonian network dynamics (Madadi Asl et al., 2022), i.e., physiological states (weak synchrony, weak connectivity) as opposed to pathological states (strong synchrony, strong connectivity) (Popovych and Tass, 2012; Lourens et al., 2015; Madadi Asl et al., 2018c). When STDP mediates the evolution of the network connections, the correlated neurons that causally fire together in a synchronized manner (with small time difference between their spikes) form stronger synapses than those uncorrelated neuronal groups that fire with large time differences in a desynchronized manner which is due to the competitive nature of STDP (Song et al., 2000). Accordingly, strong synapses between neurons can further synchronize their firing activity, whereas the activity of loosely connected neurons remains relatively desynchronized (Kozloski and Cecchi, 2010). In this way, STDP leads to the coevolution of the activity-connectivity patterns through a feedback loop in plastic networks (Lubenov and Siapas, 2008; Aoki and Aoyagi, 2009; Gilson et al., 2009; Kozloski and Cecchi, 2010; Knoblauch et al., 2012; Madadi Asl et al., 2017).

To test our hypothesis, we considered a simple model of GPe-STN network where the inter-population pallido-subthalamic synapses were modified according to an inhibitory STDP (iSTDP) rule (Woodin et al., 2003; Vogels et al., 2011). We set the network in a normal state and a PD state by adjusting the current applied to GPe and STN. Then, we explored how iSTDP can reshape GPe-STN connectivity in normal and PD state based on the uncorrelated and correlated neuronal activity in a reciprocally connected network model of GPe-STN. Particularly, uncorrelated firing of the GPe and STN neurons leads to the weakening of GPe-STN synapses due to iSTDP, establishing a loosely connected network in normal state. However, in PD state GPe-STN synapses are abnormally strengthened due to the correlated GPe-STN firing, further promoting pathologically synchronized activity in the network which is a hallmark of PD (Hammond et al., 2007).

In addition, transmission delays critically affect the emergent structure and dynamics in recurrent networks with plastic synapses (Lubenov and Siapas, 2008; Madadi Asl et al., 2017), and give rise to mutistable network dynamics (Madadi Asl et al., 2018a), i.e., coexistence of qualitatively different stable attractor states characterized by strong neuronal synchronization and strong synaptic connectivity (pathological states), and weak synchrony with weak synapses (physiological states). To further investigate the effect of delays on the dynamics and structure of the GPe-STN network in normal and PD state, we introduced transmission delays between GPe and STN and showed that inter-population delays lead to the bistability between pathological states (strong synchrony, strong connectivity) and more physiologically favored states (weak synchrony, weak connectivity). This bistability emerges due to the delay-induced shifting of spike time differences at synapses that regulates the outcome of iSTDP (Kozloski and Cecchi, 2010; Knoblauch et al., 2012; Babadi and Abbott, 2013; Madadi Asl et al., 2018b). Our results show that iSTDP may stabilize physiological and pathological patterns of neuronal activity and synaptic connectivity in the parkinsonian GPe-STN network depending on the strength of inputs and the range of transmission delays.

## Materials and Methods

### Neuron and Network Model

We used a simple model of interconnected GPe-STN network shown in Figure 1A. The input of striatum toward GPe (via the indirect pathway) and the input of cortex to STN (via the hyperdirect pathway) were simulated as a constant current. The GPe-STN network was constructed by assuming *N* = 100 randomly connected neurons within GPe and STN, as schematically shown in Figure 1B. Connection probability within each nucleus was 20%, whereas the two populations were sparsely connected to each other in a random manner with a 10% probability.

The membrane potential dynamics of GPe/STN neurons is described by a single-compartment conductance-based model introduced by Terman and colleagues (Terman et al., 2002), as follows:

$$C_{m}\frac{dV_{i}}{dt}=-I_{L}-I_{K}-I_{\text{Na}}-I_{T}-I_{\text{Ca}}-I_{\text{AHP}}-I_{\text{syn}}+I_{\text{GPe/STN}},$$ (1)

where *C*_m_ = 1 pF/*μm* ^2^ is the membrane capacitance, and *I*_GPe/STN_ is the current applied to GPe/STN. The leak current (*I*_L_), potassium current (*I*_K_), sodium current (*I*_Na_) and high-threshold calcium current (*I*_Ca_) are described by Hodgkin-Huxley-type equations:

$$\begin{array}{c} I_{L}\left(V\right)=g_{L}\left(V-V_{L}\right),\\ I_{K}\left(V\right)=g_{K}n^{4}\left(V-V_{K}\right),\\ I_{\text{Na}}\left(V\right)=g_{\text{Na}}m_{\infty }^{3}\left(V\right)h\left(V-V_{\text{Na}}\right),\\ I_{\text{Ca}}\left(V\right)=g_{\text{Ca}}s_{\infty }^{2}\left(V\right)\left(V-V_{\text{Ca}}\right), \end{array}$$ (2)

that are identical for both GPe and STN neurons. The low-threshold T-type calcium current (*I*_T_) is defined differently for GPe and STN neurons:

$$\begin{array}{c} GPe:I_{T}\left(V\right)=g_{T}a_{\infty }^{3}\left(V\right)r\left(V-V_{\text{Ca}}\right),\\ STN:I_{T}\left(V\right)=g_{T}a_{\infty }^{3}\left(V\right)b_{\infty }^{2}\left(r\right)\left(V-V_{\text{Ca}}\right), \end{array}$$ (3)

where *g*_X_ and *V*_X_ with $X\in \left\{L,K,Na,Ca\right\}$ are the maximal conductance and reversal potential of each current, respectively. The first-order kinetics of slowly operating gating variables (i.e., *n*, *h*, *r*) obeys the following differential equation:

$$\frac{dX}{dt}=\phi_{X}\left(\frac{X_{\infty }\left(V\right)-X}{\tau_{X}\left(V\right)}\right);\;X\in \left\{n,h,r\right\},$$ (4)

where *ϕ*_*X*_ is the scaling time constant of the variable *X*. The voltage-dependent time constant of the variable *X* can be written as follows:

$$\tau_{X}\left(V\right)=\tau_{X}^{0}+\frac{\tau_{X}^{1}}{1+\exp\left(-\left(V-\theta_{X}^{\tau }\right)/\sigma_{X}^{\tau }\right)};\;X\in \left\{n,h,r\right\},$$ (5)

where $\theta_{X}^{\tau }$ denotes the voltage at which the time constant is midway between its maximum and minimum values, and $\sigma_{X}^{\tau }$ represents the slope factor for the voltage dependence of the time constant. The steady-state voltage dependence of all gating variables is given by:

$$X_{\infty }\left(V\right)={\left[1+\exp\left(-\left(V-\theta_{X}\right)/\sigma_{X}\right)\right]}^{-1};\;X\in \left\{n,m,h,a,r,s\right\},$$ (6)

where *θ*_*X*_ is the half activation/inactivation voltage and *σ*_*X*_ is the slope factor. The T-type current inactivation variable (*b*), however, is described as follows:

$$b_{\infty }\left(r\right)={\left[1+\exp\left(\left(r-\theta_{b}\right)/\sigma_{b}\right)\right]}^{-1}-{\left[1+\exp\left(-\theta_{b}/\sigma_{b}\right)\right]}^{-1}.$$ (7)

The calcium-activated, voltage-independent afterhyperpolarization (AHP) potassium current (*I*_AHP_) is defined as follows:

$$I_{\text{AHP}}=g_{\text{AHP}}\left(V-V_{K}\right)\left(\frac{\left[Ca\right]}{\left[Ca\right]+k_{1}}\right),$$ (8)

where *g*_AHP_ is the maximal conductance and *k*_1_ is the dissociation constant of the calcium-dependent AHP current. The intracellular concentration of calcium ions ([Ca]) is governed by the following first-order differential equation:

$$\frac{d\left[Ca\right]}{dt}=\epsilon \left(-I_{\text{Ca}}-I_{T}-k_{\text{Ca}}\left[Ca\right]\right),$$ (9)

where *ϵ* is a constant that describes the effects of buffers, cell volume and the molar charge of calcium, and *k*_Ca_ denotes the calcium pump rate constant.

The synaptic current comprises two terms within and between neuronal populations, i.e., $I_{\text{syn}}=\sum_{j}I_{ij}^{kk}+\sum_{j}I_{ij}^{kl}$ with $k,l\in \left\{GPe,STN\right\}$ . $I_{ij}^{kl}=\sum_{j}g_{ij}^{kl}\left(V_{i}-V_{\text{syn}}^{kl}\right)\;s_{ij}^{kl}\left(t-\tau_{kl}\right)$ represents the intra-population synaptic current (*k* = *l*) within GPe/STN or the inter-population synaptic current (*k* ≠ *l*) between GPe and STN, i.e., from the presynaptic neuron *j* of population *l* to the postsynaptic neuron *i* of population *k*. $g_{ij}^{kl}$ is the corresponding synaptic strength, *V*_*i*_ is the membrane potential of the postsynaptic neuron and $V_{\text{syn}}^{kl}$ is the corresponding synaptic reversal potential. *τ*_*kl*_ denotes the transmission delay between the GPe and STN neurons perceived at the synapse. The synaptic variable *s*_*ij*_(*t*) obeys the following first-order kinetics:

$$\frac{ds_{ij}}{dt}=\alpha H_{\infty }\left(V_{j}-\theta_{g}\right)\left(1-s_{ij}\right)-\beta s_{ij},$$ (10)

where *V*_*j*_ is the membrane potential of the presynaptic neuron, *α* and *β* are the opening and closing rates of channels, respectively, and *H*_*∞*_ is given by:

$$H_{\infty }\left(V\right)={\left[1+\exp\left(-\left(V-\theta_{g}^{H}\right)/\sigma_{g}^{H}\right)\right]}^{-1}.$$ (11)

The numerical values of parameters used in our simulations are given in Tables 1, 2.

**TABLE 1:** Parameters used for maximal conductances (*g*_X_ in nS/*μ*m^2^), neuronal and synaptic reversal potentials (*V*_X_ in mV), and calcium dynamics for GPe and STN neuron models.

Cell	*g*_L_	*g*_K_	*g*_Na_	*g*_T_	*g*_Ca_	*g*_AHP_	*V*_L_	*V*_K_	*V*_Na_	*V*_Ca_	*V*_syn_	*ϵ*	*k*_Ca_	*k*_1_
GPe	0.1	30	120	0.5	0.15	30	−55	−80	55	120	0	1 × 10^–4^	20	30
STN	2.25	45	37.5	0.5	0.5	9	−60	−80	55	140	−85	5 × 10^–5^	22.5	15

**TABLE 2:** Kinetic parameters used for GPe and STN neuron models.

Cell	*X*	*θ*_*X*_	*σ*_*X*_	$\tau_{X}^{0^{a}}$	$\tau_{X}^{1^{a}}$	$\theta_{X}^{\tau }$	$\sigma_{X}^{\tau }$	*ϕ*_*X*_
GPe	*n*	−50	14	0.05	0.27	−40	−12	0.05
	*m*	−37	10	−	−	−	−	−
	*h*	−58	−12	0.05	0.27	−40	−12	0.05
	*a*	−57	2	−	−	−	−	−
	*r*	−70	−2	30	−	−	−	1
	*s*	−35	2	−	−	−	−	−
	−	*θ*_*g*_	$\theta_{g}^{H}$	$\sigma_{g}^{H}$	*α* ^b^	*β* ^b^	−	−
	−	20	−57	2	2	0.08	−	−
STN	*n*	−32	8	1	100	−80	−26	0.75
	*m*	−30	15	−	−	−	−	−
	*h*	−39	−3.1	1	500	−57	−3	0.75
	*a*	−63	7.8	−	−	−	−	−
	*r*	−67	−2	40	17.5	68	−2.2	0.2
	*s*	−39	8	−	−	−	−	−
	−	*θ*_*g*_	$\theta_{g}^{H}$	$\sigma_{g}^{H}$	*α* ^b^	*β* ^b^	*θ*_*b*_	*σ*_*b*_
	−	30	−39	8	5	1	0.4	−0.1

a Units in ms.

b Units in ms^−1^.

### Inhibitory Spike-Timing-Dependent Plasticity

In the GPe-STN network model, the STN-GPe excitatory synapses were assumed to be static (see Figure 1B), whereas the GPe-STN inhibitory synaptic connections in the pallido-subthalamic pathway (represented with strength *g*_SG_) were modified based on the following symmetric iSTDP profile (shown in Figure 3A) (Woodin et al., 2003; Vogels et al., 2011):

$$\Delta g_{\text{SG}}=\eta \;\left(\exp\left(-|\Delta t|/\tau \right)-\alpha \right),$$ (12)

where *η* is the learning rate, *τ* is the decay time constant of the exponential function and *α* is the depression factor. Δ*t* = *t*_post_ − *t*_pre_ is the time lag between pre- and postsynaptic spikes.

The synaptic strengths were updated by an additive rule at each step of the simulation, i.e., *g* → *g* + Δ*g*. The value of the synaptic strengths was confined in the range [*g*_min_, *g*_max_] ∈ [0.0, 0.5] nS/*μ*m^2^. The synaptic strengths were set to *g*_min_ (*g*_max_) *via* hard bound saturation constraint once they crossed the lower (upper) bound of their allowed range.

### Synchronized Dynamics

Rhythmic activity of GPe and STN is reflected in well-pronounced oscillations of their local field potential (LFP) which is an indicator of synchronized neuronal dynamics, defined as follows:

$$LFP\left(t\right)=N^{-1}\overset{N}{\underset{j=1}{\sum }}s_{j}\left(t\right),$$ (13)

where *s*(*t*) is the synaptic variable introduced in Eq. 10, and *N* is the number of neurons.

We also define an order parameter *r*(*t*) for the network of GPe and STN neurons ranging between 0 and 1 that measures the degree to which the system is synchronized (Kuramoto, 1984):

$$r\left(t\right)=N^{-1}\overset{N}{\underset{j=1}{\sum }}e^{i\phi_{j}\left(t\right)},$$ (14)

where *N* is the number of neurons and *ϕ*(*t*) is the phase of individual neurons. For the bursting neurons, the phase evolution was considered from consecutive bursts, i.e., between the first spike of *n*th burst and the first spike of the (*n* + 1)-th burst evaluated by a phase change of 2*π*.

## Results

### Firing Properties of GPe and STN Neurons

**FIGURE 2:**
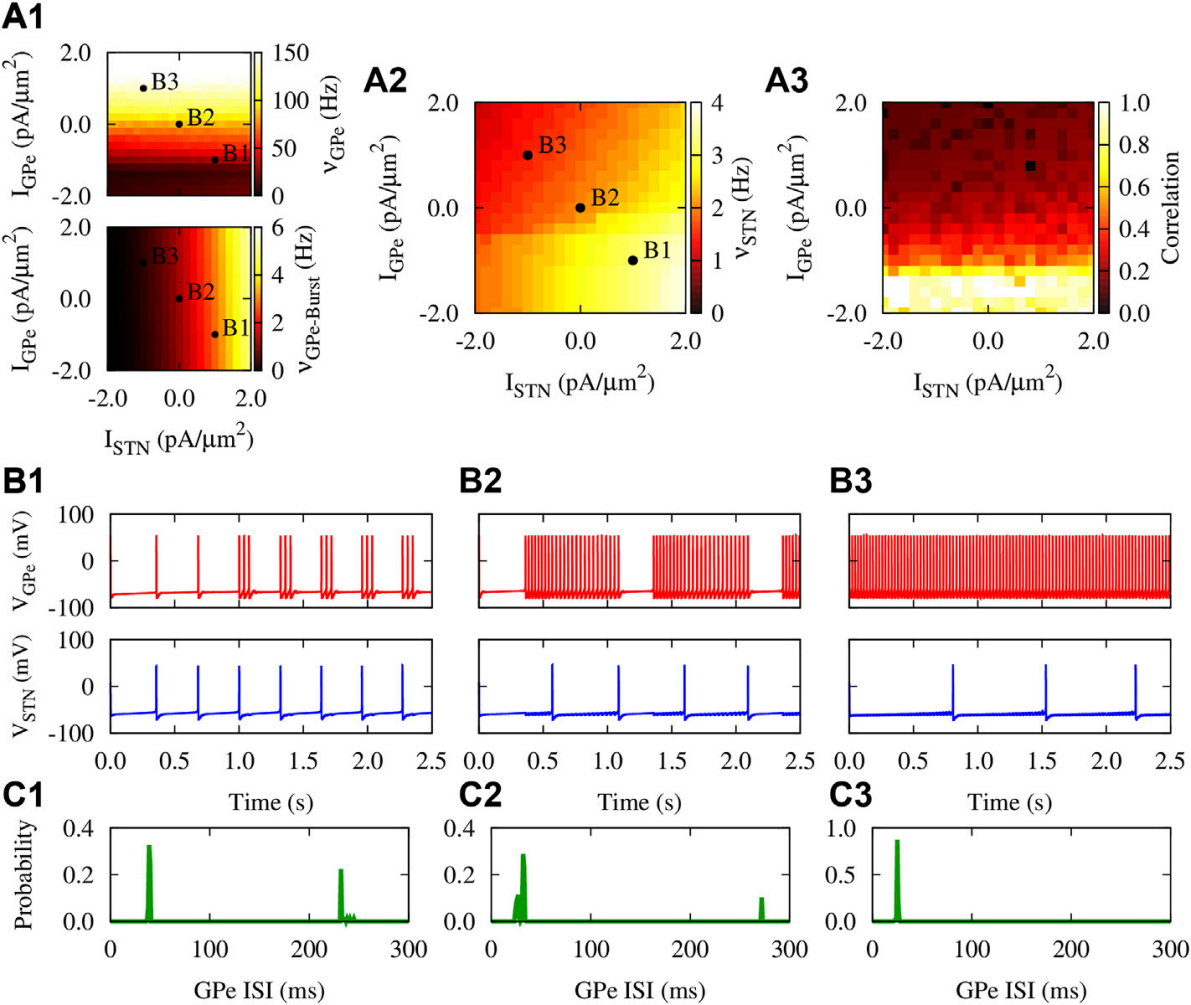
Firing properties of the GPe-STN motif with static synapses. **(A1–A3)** Mean firing rate [**(A1)**, top] and burst frequency [**(A1)**, bottom] of the GPe neuron, mean firing rate of the STN neuron **(A2)**, and the pairwise correlation between their spiking activity **(A3)** as a function of applied currents when *g*_SG_ = *g*_GS_ = 0.25 nS/*μ*m^2^. The burst frequency for the GPe neuron was calculated by considering the first spike in each burst. **(B1–B3)** Three examplary time courses of the membrane potential of reciprocally connected GPe (top) and STN (bottom) neurons subjected to different applied currents: **(B1)** *I*_GPe_ = −1.0 pA/*μ*m^2^, *I*_STN_ = 1.0 pA/*μ*m^2^ **(B2)** *I*_GPe_ = *I*_STN_ = 0.0 pA/*μ*m^2^. **(B3)** *I*_GPe_ = 1.0 pA/*μ*m^2^, *I*_STN_ = −1.0 pA/*μ*m^2^ **(C1–C3)** The distribution of ISIs of the firing activity of the GPe neuron shown in **(B1–B3)** (top), respectively.

Firing properties of the GPe-STN motif comprising an inhibitory GPe neuron reciprocally connected to an excitatory STN neuron are shown in Figure 2 under hyperpolarizing, zero, and depolarizing currents applied to GPe and STN. The mean firing rate of the GPe neuron is notably increased when the GPe input (*I*_GPe_) is shifted from a hyperpolarizing current to zero and, then, to a depolarizing current (Figure 2A1, top, points B1-B3). In this case, the firing mode of the GPe neuron is transformed from the continuous cluster firing to the episodic firing and, then, to the continuous firing mode (Terman et al., 2002), as shown in Figures 2B1–B3 (top). This behavior is also reflected in the unimodal and bimodal interspike interval (ISI) distribution of the GPe firing activity shown in Figures 2C1–C3. Furthermore, the burst frequency for the GPe neuron in Figure 2A1 (bottom) shows the transition from the burst firing mode (e.g., point B1) to the continuous firing mode (e.g., point B3) as the applied currents are changed.

In this way, elevated activity of the GPe neuron further inhibits the STN neuron, resulting in the reduced mean firing rate of the STN cell, as is shown in Figures 2B1–B3 (bottom). However, increasing the STN input (*I*_STN_) led to an increased mean firing rate of STN neuron as shown in Figures 2A2, 2B3–B1 (bottom). Moreover, Figure 2A3 shows that when a hyperpolarizing current is applied to GPe, the pairwise correlation between the spiking activity of GPe and STN neurons is notably enhanced compared to a depolarizing applied current. This mimics the PD condition where the striato-pallidal inhibition is profoundly increased, leading to abnormally correlated GPe-STN firing activity.

### The GPe-STN Network Mediated by iSTDP

**FIGURE 3:**
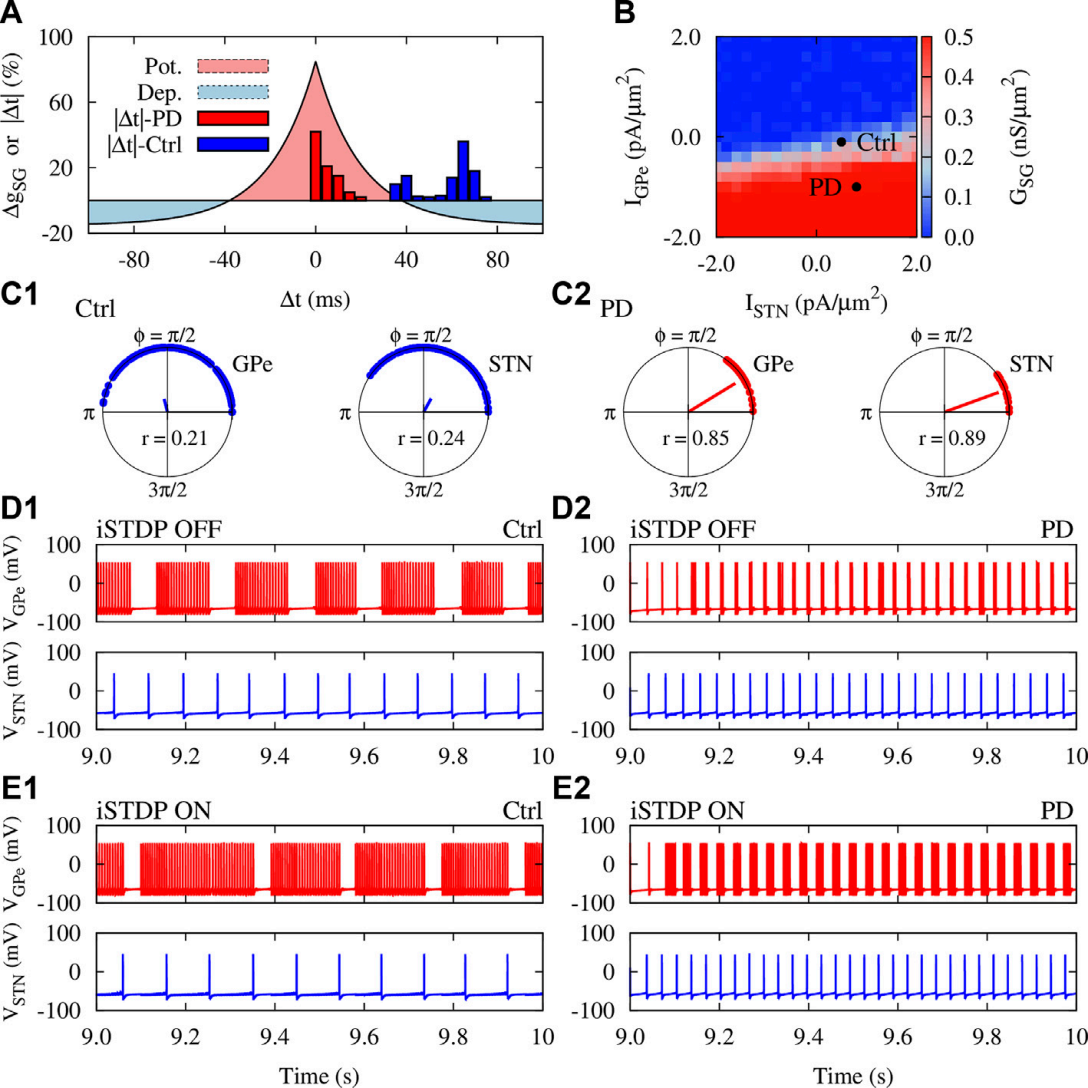
iSTDP shapes neuronal synchrony and inter-population coupling in the GPe-STN network. **(A)** The iSTDP profile described by Eq. 12 with *η* = 0.005 nS/*μ*m^2^, *τ* = 20 ms and *α* = 0.15. Blue (red) boxes show the distribution of time lags between the firing of neurons obtained from numerical simulations for control (PD) condition marked in panel **(B) (B)** Steady-state mean coupling adjusted by iSTDP in the pallido-subthalamic pathway (*G*_SG_) for different mean currents applied to GPe and STN. Initial strength of individual synapses was picked from a normal distribution around 0.25 nS/*μ*m^2^. **(C1,C2)** Polar distribution of the phases of the GPe (left) and STN (right) neurons in control **(C1)** and PD **(C2)** condition. The radial bar denotes the order parameter defined in Eq. 14, where its value is indicated within each panel. **(D1,D2)** Examplary time course of the membrane potential of randomly chosen GPe (#68) and STN (#43) neurons in control **(D1)** and PD **(D2)** condition when STDP was OFF. Ctrl: *I*_GPe_ = −0.1 pA/*μ*m^2^, *I*_STN_ = 0.5 pA/*μ*m^2^ and PD: *I*_GPe_ = −1.0 pA/*μ*m^2^, *I*_STN_ = 0.8 pA/*μ*m^2^ (marked in panel B). **(E1,E2)** Same as **(D1)** and **(D2)**, but when iSTDP was turned ON.

To inspect how the presence of synaptic plasticity can affect emergent structure and dynamics of the GPe-STN network, we assumed that synapses in the GPe-STN pathway are modified according to the iSTDP rule given by Eq. 12, as shown in Figure 3A. This symmetric iSTDP profile is a generic form of inhibitory synaptic plasticity observed in hippocampus (Woodin et al., 2003) and cortex (D’amour and Froemke, 2015). Unlike the asymmetric shape of the classical STDP rule (Gerstner et al., 1996; Markram et al., 1997; Bi and Poo, 1998), where the order of spike pairs (pre-post pairing or post-pre pairing) determines long-term potentiation (LTP) vs. long-term depression (LTD.) of synapses, modification of inhibitory synapses takes place trough a temporally symmetric profile, i.e., when the difference between spike timings of the two neurons (time lag) is smaller than a given time lag, |Δt*| = −*τ* ln*α* in Eq. 12, the corresponding synapse is strengthened (Figure 3A, red region), otherwise the synapse is weakened (Figure 3A, blue region).

To construct the GPe-STN network, we considered *N* = 100 randomly connected neurons in each nucleus, as schematically shown in Figure 1B. Only synaptic connections from GPe to STN were subjected to iSTDP and STN-GPe synapses were assumed to be static. To avoid a biased initial setting, the value of both GPe-STN and STN-GPe synaptic strengths were randomly chosen from a normal distribution whose mean was set in the middle of the allowed range, i.e., *g*_SG_ (*t* = 0) = *g*_GS_ = 0.25 nS/*μ*m^2^. The steady-state mean GPe-STN synaptic coupling mediated by iSTDP is depicted in Figure 3B which shows transitions between weak (blue) and strong (red) connectivity regimes as a function of currents applied to GPe and STN.

The findings of experimental PD models suggest that both striato-pallidal (Lemos et al., 2016) and cortico-subthalamic (Chu et al., 2017) inputs are augmented in the PD state. Therefore, we computationally discriminated normal (control) and PD state based on the currents applied to GPe and STN which were chosen from a normal distribution whose mean was Ctrl: *I*_GPe_ = −0.1 pA/*μ*m^2^, *I*_STN_ = 0.5 pA/*μ*m^2^ and PD: *I*_GPe_ = −1.0 pA/*μ*m^2^, *I*_STN_ = 0.8 pA/*μ*m^2^ (marked in Figure 3B).

The time difference between spike timings of the GPe-STN pairs in the network for the marked ctrl (PD) parameters in Figure 3B are shown in Figure 3A by blue (red) bars. In control condition, the spiking activity of GPe and STN neurons is relatively uncorrelated with large time lags between spike pairs leading to the dominance of LTD. over LTP (see Figure 3A, blue bars/blue region). This ultimately results in the weakening of GPe-STN synaptic strengths and, the mean GPe-STN coupling, as shown in Figure 3B (ctrl). In PD state, however, the activity of GPe and STN neurons is more correlated leading to small lags between spiking timings of neurons (see Figure 3A, red bars/red region). As a result, GPe-STN synapses are potentiated as it is reflected in the mean GPe-STN coupling shown in Figure 3B (PD). In addition, the current conditions for control and PD states make a system transition from non-synchronized activity states (Figure 3C1) to more synchronized states (Figure 3C2) as shown by the distribution of the phases of neurons within each population and the corresponding order parameter.

Interestingly, iSTDP not only modulated the firing frequency of GPe neurons, but also changed their firing pattern so that the number of spikes per burst was increased. Figure 3D1, E1 (top) shows that, on average, the number of spikes per burst in the #68 GPe neuron is increased in control condition when iSTDP is turned ON (i.e., number of spikes/burst: 18 ± 1.4, iSTDP OFF vs. 30 ± 2.1, iSTDP ON). In PD state, the number of spikes per burst is also slightly increased due to the presence of iSTDP as shown in Figure 3D2, E2 (i.e., number of spikes/burst: 2 ± 0.8, iSTDP OFF vs. 5 ± 0.3, iSTDP ON). Unlike GPe neurons, only the firing rate of STN neurons was affected by iSTDP. For instance, iSTDP decreased the mean firing rate of the #43 STN neuron in control condition (cf. Figure 3D1, E1, bottom), however, the mean firing rate was increased in PD condition when iSTDP was turned ON (cf. Figures 3D2, E2, bottom).

### Normal and Parkinsonian Networks Mediated by iSTDP

**FIGURE 4:**
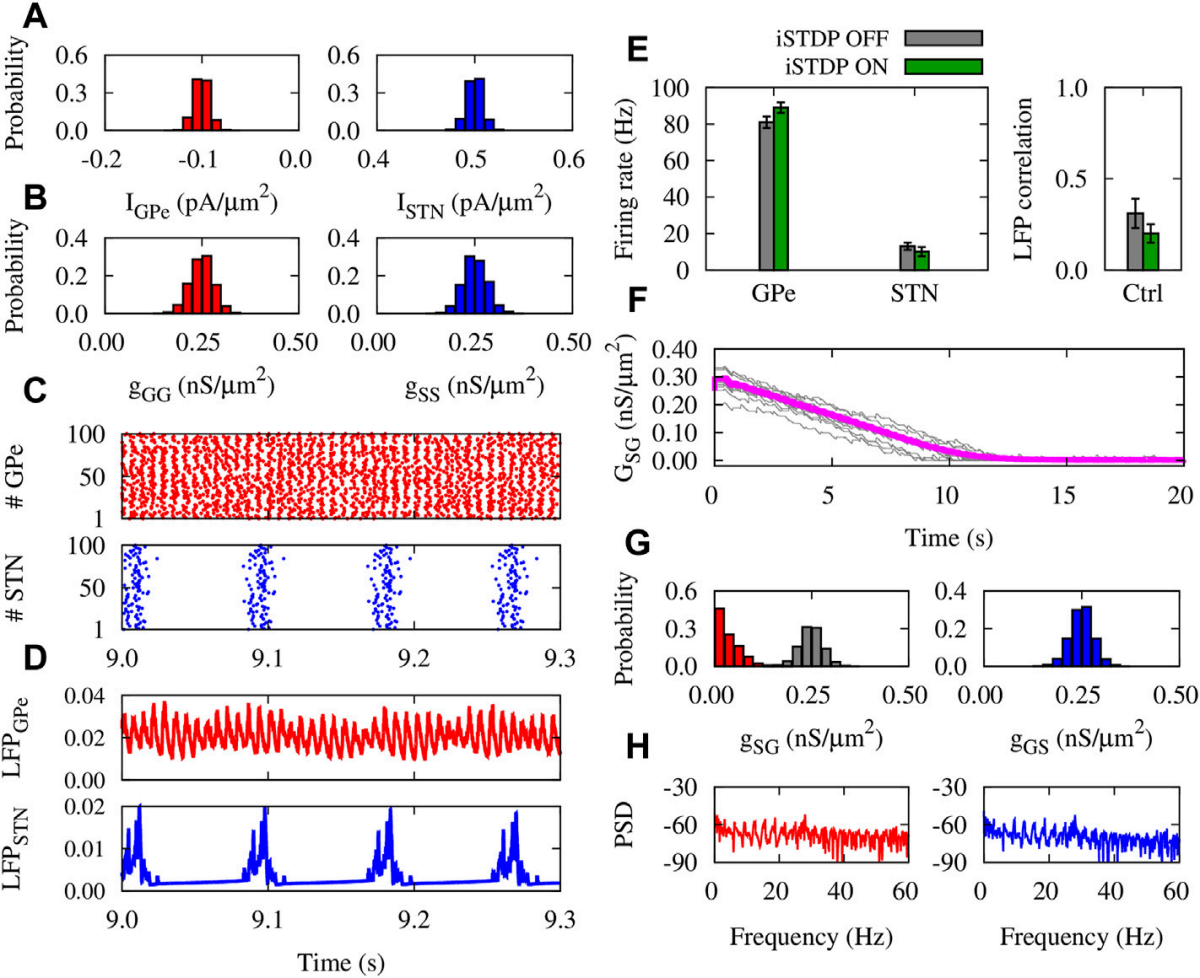
Properties of the GPe-STN network mediated by iSTDP in control condition. **(A)** Normal distribution of currents applied to GPe (left) and STN (right) around *I*_GPe_ = −0.1 pA/*μ*m^2^ and *I*_STN_ = 0.5 pA/*μ*m^2^. **(B)** Normal distribution of GPe-GPe (left) and STN-STN (right) synaptic strengths around 0.25 nS/*μ*m^2^. **(C)** Raster plots of GPe (top) and STN (bottom). **(D)** LFP of GPe (top) and STN (bottom) defined in Eq. 13. **(E)** Mean firing rate of GPe and STN neurons (left) and the correlation between the GPe and STN LFPs (right). **(F)** Time course of the mean GPe-STN coupling (magenta) and ten randomly chosen individual synaptic strengths (grey). **(G)** Normal distribution of the initial GPe-STN (left; grey) and STN-GPe (right) synaptic strengths. Final distribution of the GPe-STN synaptic strengths is depicted in red (left). **(H)** PSD of the GPe (left) and STN (right) LFPs.

The dynamics and structure of the GPe-STN network subjected to iSTDP in normal condition is shown in Figure 4. The currents applied to GPe and STN neurons (Figure 4A), and the initial synaptic weights within GPe and STN (Figure 4B) were chosen from a normal distribution. Raster plots (Figure 4C) and LFPs (Figure 4D) of GPe (top) and STN (bottom) show that the activity of neurons is weakly synchronized. iSTDP increased (decreased) the mean firing rate of GPe (STN) compared to iSTDP OFF state, i.e., GPe: 91 ± 2.5 Hz (iSTDP ON) vs. 80 ± 2.1 Hz (iSTDP OFF), and STN: 10 ± 0.5 Hz (iSTDP ON) vs. 13 ± 0.7 Hz (iSTDP ON), as shown in Figure 4E (left).

iSTDP suppressed correlation between GPe and STN LFP (Figure 4E, right), promoting normal (uncorrelated) GPe-STN activity. Uncorrelated neuronal activity leads to the down-regulation (depression) of the mean synaptic coupling by iSTDP in the GPe-STN pathway (Figure 4F), as it is reflected in the final distribution (red) of the synaptic strengths in comparison to the initial distribution (grey) shown in Figure 4G (left). iSTDP shapes more physiologically favored activity-connectivity patterns (i.e., weak neuronal synchrony and weak synaptic connectivity), where sharp beta band picks are absent in the power spectrum density (PSD) of GPe and STN activity (see Figure 4H).

**FIGURE 5:**
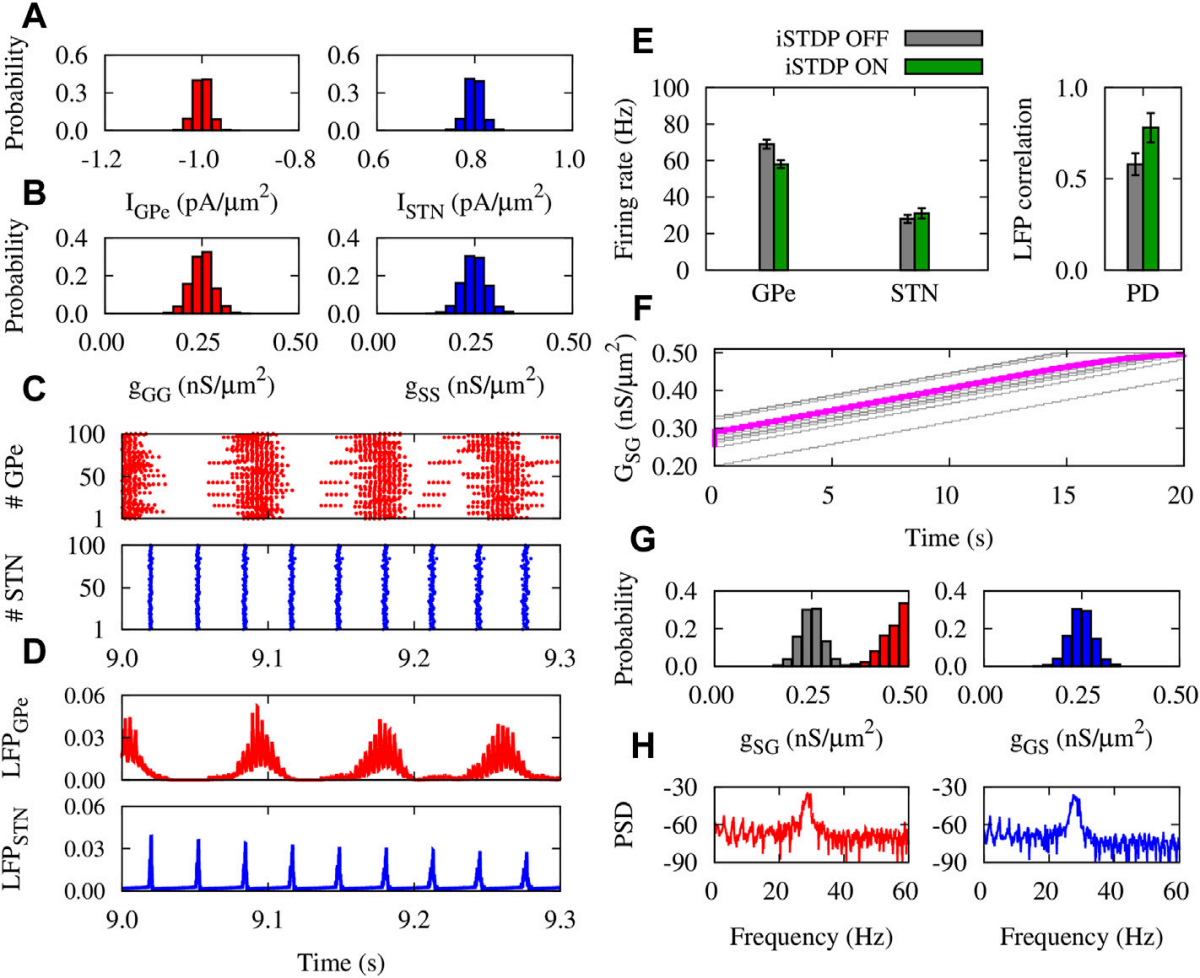
Properties of the GPe-STN network mediated by iSTDP in PD condition. **(A)** Normal distribution of currents applied to GPe (left) and STN (right) around *I*_GPe_ = −1.0 pA/*μ*m^2^ and *I*_STN_ = 0.8 pA/*μ*m^2^. **(B)** Normal distribution of GPe-GPe (left) and STN-STN (right) synaptic strengths around 0.25 nS/*μ*m^2^. **(C)** Raster plots of GPe (top) and STN (bottom). **(D)** LFP of GPe (top) and STN (bottom). **(E)** Mean firing rate of GPe and STN neurons (left) and the correlation between the GPe and STN LFPs (right). **(F)** Time course of the mean GPe-STN coupling (magenta) and ten randomly chosen individual synaptic strengths (grey). **(G)** Normal distribution of the initial GPe-STN (left; grey) and STN-GPe (right) synaptic strengths. Final distribution of the GPe-STN synaptic strengths is depicted in red (left). **(H)** PSD of the GPe (left) and STN (right) LFPs.

In PD condition, only the mean value of the normal distribution of currents applied to GPe and STN neurons was chosen differently from normal condition (Figure 5A). The initial distributions of synaptic weights within GPe and STN were similar to normal condition (Figure 5B). Raster plots (Figure 5C) and LFPs (Figure 5D) show that GPe neurons (top) fire in a bursting manner, whereas the activity of STN neurons (bottom) is strongly synchronized. In this case, the mean firing rate of GPe (STN) neurons was decreased (increased), in comparison to the control condition (cf. Figures 4E, 5E, left). iSTDP further decreased (increased) the mean firing rate of GPe (STN), as shown in Figure 5E (green), i.e., GPe: 58 ± 1.9 Hz (iSTDP ON) vs. 70 ± 2.3 Hz (iSTDP OFF), and STN: 30 ± 1.3 Hz (iSTDP ON) vs. 24 ± 1.1 Hz (iSTDP OFF).

Notably, iSTDP increased the correlation between GPe and STN LFP (Figure 5E, right), promoting pathologically correlated GPe-STN activity which is a hallmark of PD (Hammond et al., 2007). Abnormally correlated neuronal activity leads to the up-regulation (potentiation) of the inter-population mean synaptic coupling by iSTDP (Figure 5F), so that the final (red) synaptic strengths saturated to their maximum allowed value compared to the initial distribution (grey) shown in Figure 5G (left). In PD condition, iSTDP supports pathological activity-connectivity patterns (i.e., strong neuronal synchrony and strong synaptic connectivity), characterized by sharp beta band picks in the PSD of GPe and STN activity (see Figure 5H).

### Delay Dependency of Emergent Activity-Connectivity Patterns

**FIGURE 6:**
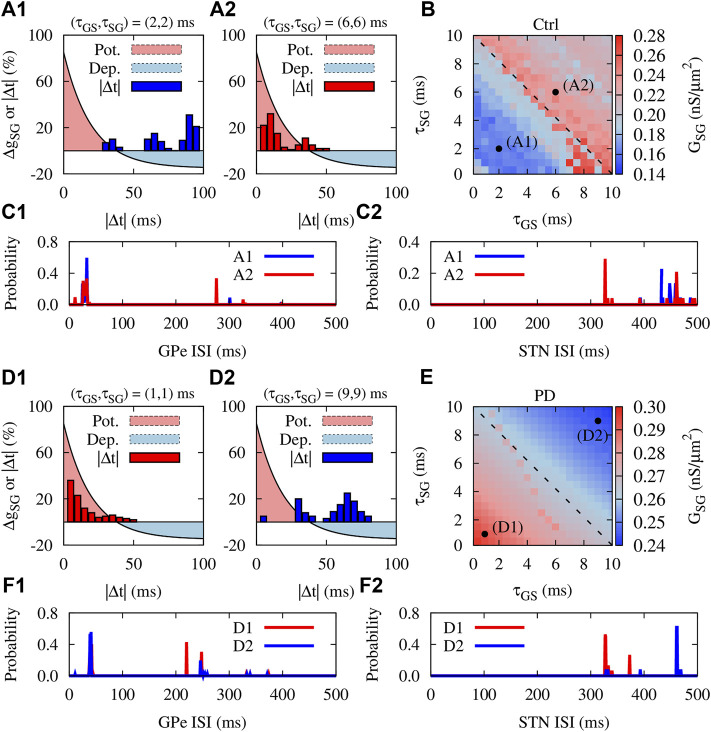
Delay dependency of activity-connectivity states in the GPe-STN network with iSTDP. Control condition: **(A1,A2)** The iSTDP profile described by Eq. 4 depicted for positive time lags. Blue **(A1)** and red **(A2)** bars show the distribution of time lags between the firing of GPe and STN neurons obtained from numerical simulations for different transmission delays indicated above each panel. **(B)** Steady-state mean synaptic coupling in the pallido-subthalamic pathway for different feedforward and feedback transmission delays. Dashed line indicates *τ*_SG_ = −*τ*_GS_ + 10 ms. **(C1,C2)** The distribution of ISIs of the GPe **(C1)** and STN **(C2)** neurons in control condition for the range of delays used in panels A1 and A2. **(D1–F2)** Same as **(A1–C2)**, but for PD condition.

The findings of experimental studies in rats (Fujimoto and Kita, 1993) and monkeys (Kita et al., 2005) revealed that the delay in transmission from GPe to STN (and vice versa) can assume values around a few milliseconds. Accordingly, computational modeling studies considered similar values for the transmission delays between GPe and STN (Holgado et al., 2010). Previously, it has been shown that transmission delays can shape multistable dynamics in neuronal networks with STDP (Madadi Asl et al., 2018a, b), i.e., qualitatively different stable states of activity and connectivity may emerge due to the interplay between delays and STDP. To address how the presence of transmission delays can modulate the emergent neuronal activity and synaptic connectivity mediated by iSTDP in normal and PD condition, we repeated our simulations for the GPe-STN network. The results are summed in Figure 6.

To inspect the potential role of delays in neuronal synchronization in the GPe-STN network due to iSTDP, we first assumed that transmission delay in the GPe-STN (*τ*_SG_) and STN-GPe (*τ*_GS_) pathways are identical and then, observed the time lag between firing of GPe and STN neurons. In control condition, when the delays were relatively small (2 ms), neurons fired in a desynchronized manner with large time lags between their spike times (Figure 6A1, blue bars). However, larger delays (6 ms) led to a more synchronized neuronal firing where neurons fired with relatively small time lags (Figure 6A1, red bars). Interestingly, when the distribution of time lags was overlaid on the iSTDP profile (Figures 6A1, A2, light red/blue region), the resultant overlap indicates that the GPe-STN synaptic connectivity can be up-/down-regulated depending on the time lag.

This is shown in Figure 6B where feedforward and feedback delays were varied up to 10 ms and the emergent steady-state mean synaptic coupling between GPe and STN was measured. Figure 6 shows a delay-induced bistability in GPe-STN synaptic connectivity due to iSTDP. By reasonably assuming that the delays are positive and are confined in the range $\left(\right.0,10\left.\right]$ ms, when *τ*_SG_ < − *τ*_GS_ + 10 ms, weak synaptic connections are achieved (Figure 6B, blue) that is reflective of large time lags between the firing of neurons, as shown in Figure 6A1, where the corresponding range of delays is marked in panel B. On the contrary, when *τ*_SG_ > − *τ*_GS_ + 10 ms the synaptic strengths are up-regulated by iSTDP to form stronger inter-population synaptic connectivity (Figure 6B, red) due to small time lags between the activity of GPe and STN neurons (point A2 refers to range of delays used in Figure 6A2). This delay-induced bistability between weak and strong connectivity regimes may be attributed to the distribution of ISIs of neurons at a given delay. As shown in Figures 6C1, C2, by changing the range of delays (as used in panels A1 and A2) the firing frequency of GPe and STN neurons as well as their firing pattern is changed, leading to changes in the inter-population synapses.

In PD condition, however, the situation is reversed. Small delays (e.g., 1 ms) led to synchronized activity of GPe and STN with small time lags between their spikes (Figure 6D1, red bars), leading to the potentiation of the synaptic strengths by iSTDP and the emergence of strong inter-population synaptic connectivity (Figure 6E, point D1). Greater delays (e.g., 9 ms) led to the desynchronized activity of GPe and STN neurons characterized by large time lags (Figure 6D2, blue bars). This ultimately led to the depression of the synaptic strengths, shaping a loosely connected GPe-STN network structure due to iSTDP (Figure 6E, point D2). Accordingly, these changes in inter-population connectivity relate to changes in the firing frequency of GPe and STN neurons as well as their firing pattern at different delays, as demonstrated by the distribution of ISIs of neurons in Figures 6F1, F2. In this way, by shaping bistable activity-connectivity states, transmission delays and iSTDP cooperate to shift the activity-connectivity patterns from physiological states (weak synchrony, weak connectivity) towards pathological states (strong synchrony, strong connectivity), and vice versa.

## Discussion

The GPe-STN synaptic transmission is significantly augmented following DA depletion in experimental PD models (Fan et al., 2012). Abnormal strengthening of GPe-STN synapses was experimentally attributed to motor cortical-driven heterosynaptic long-term potentiation (hLTP) resulted from interactions between the hyperdirect and indirect pathways (Chu et al., 2015, 2017). However, findings in animal PD models have revealed that the knockdown of STN NMDARs prevents the strengthening of GPe-STN synapses (Chu et al., 2015), suggesting that classical NMDAR-dependent LTP may be involved in this process (Madadi Asl et al., 2022).

Here, we considered a simple GPe-STN network model and computationally showed that when GPe-STN synapses are modified by inhibitory spike-timing-dependent plasticity (iSTDP), a set of parameters mimicking PD condition leads to the emergence of pathological activity-connectivity states, i.e., strong neuronal synchronization and strong synaptic connectivity between GPe and STN. On the contrary, in normal condition iSTDP stabilized physiological activity-connectivity states characterized by weak neuronal synchronization and weak synaptic connectivity. Our model neglects several biological and structural aspects of the BG circuitry. For example, both the cortex-striatum-GPe-STN and cortex-STN inputs were simply simulated as constant currents and there was no interactions between the BG, cortex and thalamus. Our model may not be able to capture the complex network interactions within the cortico-BG-thalamic circuits leading to pathological structure and dynamics in PD (Madadi Asl et al., 2022), but still can reproduce fundamental biophysical mechanisms related to pathological activity-connectivity patterns in the GPe-STN network, which is considered as the main rhythmogenesis center within the cortico-BG-thalamic network (Plenz and Kital, 1999; Bevan et al., 2002; Holgado et al., 2010).

Under normal conditions, the activity of GPe and STN neurons are poorly correlated. Therefore, the GPe-STN synapses are more likely to be weakened by iSTDP. Down-regulation of GPe-STN synaptic connectivity further promotes uncorrelated GPe-STN neuronal activity in a feedback loop. In PD condition, however, the activity of GPe and STN is strongly correlated, leading to the abnormal strengthening of GPe-STN synapses due to iSTDP. In this way, up-regulated GPe-STN synaptic connectivity promotes abnormal neuronal activity within GPe and STN. In addition, we showed that inter-population transmission delays in the iSTDP-mediated GPe-STN network lead to the bistablity between physiological and pathological activity-connectivity states. Feedforward and feedback delays in the GPe-STN network are therefore important parameters that determine different activity-connectivity states that emerge through iSTDP.

Synaptic plasticity is one of the mechanisms that mediates transitions between physiological and pathological activity-connectivity states (Lourens et al., 2015; Berner et al., 2021). However, impaired synaptic plasticity is involved in biological systems (Madadi Asl and Ramezani Akbarabadi, 2021) and several neuropsychiatric disorders (Madadi Asl et al., 2019). In PD, for instance, DA-mediated synaptic plasticity is disturbed following DA loss (Kerr and Wickens, 2001; Pawlak and Kerr, 2008), so that the temporal asymmetry of the STDP learning window required for the induction of LTP and LTD is dramatically changed (Pawlak and Kerr, 2008; Shen et al., 2008; Dupuis et al., 2013). Since neuronal activity and synaptic connectivity in plastic networks are strongly correlated, abnormal reshaping of synaptic connectivity due to impaired synaptic plasticity following DA depletion can lead to the development of pathological neuronal activity within the BG (Moran et al., 2011), further promoting pathological connectivity patterns through STDP (Madadi Asl et al., 2022). How exactly the DA-mediated STDP can affect activity-connectivity patterns in normal and pathological condition deserves to be focused on in future studies both from a computational and experimental perspective.

Finally, STDP-induced bistability between pathological states (i.e., strong neuronal synchronization, strong synaptic connectivity) and more physiologically favored states (i.e., weak neuronal synchronization, weak synaptic connectivity) could be of interest for therapeutic interventions in PD (Lourens et al., 2015; Kromer and Tass, 2020; Schwab et al., 2021). Computationally, by subtle tuning of the stimulation pattern, novel deep brain stimulation (DBS) techniques such as coordinated reset (CR) stimulation (Tass, 2003) were able to exploit STDP-induced bistability to shift the dynamics of pathological networks towards physiological attractor states (Tass and Majtanik, 2006; Hauptmann and Tass, 2009; Popovych and Tass, 2012; Kromer and Tass, 2020). This ultimately leads to an unlearning of abnormal neuronal synchrony and abnormal synaptic connectivity, so that an anti-kindling is achieved (Tass and Majtanik, 2006). The pre-clinical development and testing of CR-DBS in parkinsonian monkeys (Tass et al., 2012; Wang et al., 2016), for instance, was inspired by the predictions of these computational studies. More recently, clinical studies showed that CR-DBS can successfully induce long-lasting improvement of motor symptoms in PD patients (Syrkin-Nikolau et al., 2018; Pfeifer et al., 2021). Our results may shed light on the role of synaptic plasticity in structural and functional reorganization of the GPe-STN network during parkinsonism and, therefore, can be beneficial for the optimization and development of therapeutic stimulation strategies targeting STDP-induced changes in network connectivity.

## Data Availability

The original contributions presented in the study are included in the article/Supplementary Material, further inquiries can be directed to the corresponding author.

## References

[B1] Aoki T. Aoyagi T. (2009). Co-evolution of Phases and Connection Strengths in a Network of Phase Oscillators. Phys. Rev. Lett. 102, 034101. 10.1103/PhysRevLett.102.034101 19257356

[B2] Asadi A. Madadi Asl M. Vahabie A.-H. Valizadeh A. (2022). The Origin of Abnormal Beta Oscillations in the Parkinsonian Corticobasal Ganglia Circuits. Parkinson's Dis. 2022, 1–13. 10.1155/2022/7524066 PMC8896962 35251590

[B3] Babadi B. Abbott L. F. (2013). Pairwise Analysis Can Account for Network Structures Arising from Spike-Timing Dependent Plasticity. PLoS Comput. Biol. 9, e1002906. 10.1371/journal.pcbi.1002906 23436986 PMC3578766

[B4] Berner R. Vock S. Schöll E. Yanchuk S. (2021). Desynchronization Transitions in Adaptive Networks. Phys. Rev. Lett. 126, 028301. 10.1103/PhysRevLett.126.028301 33512200

[B5] Bevan M. Magill P. J. Terman D. Bolam J. P. Wilson C. J. (2002). Move to the Rhythm: Oscillations in the Subthalamic Nucleus-External Globus Pallidus Network. Trends Neurosci. 25, 525–531. 10.1016/s0166-2236(02)02235-x 12220881

[B6] Bi G.-q. Poo M.-m. (1998). Synaptic Modifications in Cultured Hippocampal Neurons: Dependence on Spike Timing, Synaptic Strength, and Postsynaptic Cell Type. J. Neurosci. 18, 10464–10472. 10.1523/jneurosci.18-24-10464.1998 9852584 PMC6793365

[B7] Brown P. Oliviero A. Mazzone P. Insola A. Tonali P. Di Lazzaro V. (2001). Dopamine Dependency of Oscillations between Subthalamic Nucleus and Pallidum in Parkinson's Disease. J. Neurosci. 21, 1033–1038. 10.1523/jneurosci.21-03-01033.2001 11157088 PMC6762327

[B8] Chu H.-Y. Atherton J. F. Wokosin D. Surmeier D. J. Bevan M. D. (2015). Heterosynaptic Regulation of External Globus Pallidus Inputs to the Subthalamic Nucleus by the Motor Cortex. Neuron 85, 364–376. 10.1016/j.neuron.2014.12.022 25578364 PMC4304914

[B9] Chu H.-Y. McIver E. L. Kovaleski R. F. Atherton J. F. Bevan M. D. (2017). Loss of Hyperdirect Pathway Cortico-Subthalamic Inputs Following Degeneration of Midbrain Dopamine Neurons. Neuron 95, 1306–1318. 10.1016/j.neuron.2017.08.038 28910619 PMC5679443

[B10] D’amour J. A. Froemke R. C. (2015). Inhibitory and Excitatory Spike-timing-dependent Plasticity in the Auditory Cortex. Neuron 86, 514–528. 25843405 10.1016/j.neuron.2015.03.014 PMC4409545

[B11] Day M. Wang Z. Ding J. An X. Ingham C. A. Shering A. F. (2006). Selective Elimination of Glutamatergic Synapses on Striatopallidal Neurons in Parkinson Disease Models. Nat. Neurosci. 9, 251–259. 10.1038/nn1632 16415865

[B12] DeLong M. R. Wichmann T. (2007). Circuits and Circuit Disorders of the Basal Ganglia. Arch. Neurol. 64, 20–24. 10.1001/archneur.64.1.20 17210805

[B13] Dupuis J. P. Feyder M. Miguelez C. Garcia L. Morin S. Choquet D. (2013). Dopamine-dependent Long-Term Depression at Subthalamo-Nigral Synapses Is Lost in Experimental Parkinsonism. J. Neurosci. 33, 14331–14341. 10.1523/jneurosci.1681-13.2013 24005286 PMC6618387

[B14] Fan K. Y. Baufreton J. Surmeier D. J. Chan C. S. Bevan M. D. (2012). Proliferation of External Globus Pallidus-Subthalamic Nucleus Synapses Following Degeneration of Midbrain Dopamine Neurons. J. Neurosci. 32, 13718–13728. 10.1523/jneurosci.5750-11.2012 23035084 PMC3475197

[B15] Fujimoto K. Kita H. (1993). Response Characteristics of Subthalamic Neurons to the Stimulation of the Sensorimotor Cortex in the Rat. Brain Res. 609, 185–192. 10.1016/0006-8993(93)90872-k 8508302

[B16] Galvan A. Devergnas A. Wichmann T. (2015). Alterations in Neuronal Activity in Basal Ganglia-Thalamocortical Circuits in the Parkinsonian State. Front. Neuroanat. 9, 5. 10.3389/fnana.2015.00005 25698937 PMC4318426

[B17] Galvan A. Wichmann T. (2008). Pathophysiology of Parkinsonism. Clin. Neurophysiol. 119, 1459–1474. 10.1016/j.clinph.2008.03.017 18467168 PMC2467461

[B18] Gerstner W. Kempter R. van Hemmen J. L. Wagner H. (1996). A Neuronal Learning Rule for Sub-millisecond Temporal Coding. Nature 383, 76–78. 10.1038/383076a0 8779718

[B19] Gilson M. Burkitt A. N. Grayden D. B. Thomas D. A. van Hemmen J. L. (2009). Emergence of Network Structure Due to Spike-timing-dependent Plasticity in Recurrent Neuronal Networks Iv. Biol. Cybern. 101, 427–444. 10.1007/s00422-009-0346-1 19937070

[B20] Hammond C. Bergman H. Brown P. (2007). Pathological Synchronization in Parkinson's Disease: Networks, Models and Treatments. Trends Neurosci. 30, 357–364. 10.1016/j.tins.2007.05.004 17532060

[B21] Hauptmann C. Tass P. A. (2009). Cumulative and After-Effects of Short and Weak Coordinated Reset Stimulation: a Modeling Study. J. Neural Eng. 6, 016004. 10.1088/1741-2560/6/1/016004 19141875

[B22] Holgado A. J. N. Terry J. R. Bogacz R. (2010). Conditions for the Generation of Beta Oscillations in the Subthalamic Nucleus-Globus Pallidus Network. J. Neurosci. 30, 12340–12352. 10.1523/jneurosci.0817-10.2010 20844130 PMC6633459

[B23] Kerr J. N. D. Wickens J. R. (2001). Dopamine D-1/d-5 Receptor Activation Is Required for Long-Term Potentiation in the Rat Neostriatum *In Vitro* . J. Neurophysiology 85, 117–124. 10.1152/jn.2001.85.1.117 11152712

[B24] Kita H. Tachibana Y. Nambu A. Chiken S. (2005). Balance of Monosynaptic Excitatory and Disynaptic Inhibitory Responses of the Globus Pallidus Induced after Stimulation of the Subthalamic Nucleus in the Monkey. J. Neurosci. 25, 8611–8619. 10.1523/jneurosci.1719-05.2005 16177028 PMC6725523

[B25] Knoblauch A. Hauser F. Gewaltig M.-O. Körner E. Palm G. (2012). Does Spike-timing-dependent Synaptic Plasticity Couple or Decouple Neurons Firing in Synchrony? Front. Comput. Neurosci. 6, 55. 10.3389/fncom.2012.00055 22936909 PMC3424530

[B26] Kozloski J. Cecchi G. A. (2010). A Theory of Loop Formation and Elimination by Spike Timing-dependent Plasticity. Front. Neural Circuits 4, 7. 10.3389/fncir.2010.00007 20407633 PMC2856591

[B27] Kromer J. A. Tass P. A. (2020). Long-lasting Desynchronization by Decoupling Stimulation. Phys. Rev. Res. 2, 033101. 10.1103/physrevresearch.2.033101

[B28] Kühn A. A. Kupsch A. Schneider G.-H. Brown P. (2006). Reduction in Subthalamic 8-35 Hz Oscillatory Activity Correlates with Clinical Improvement in Parkinson's Disease. Eur. J. Neurosci. 23, 1956–1960. 10.1111/j.1460-9568.2006.04717.x 16623853

[B29] Kuramoto Y. (1984). Chemical Oscillations, Waves, and Turbulence. Berlin: Springer.

[B30] Lemos J. C. Friend D. M. Kaplan A. R. Shin J. H. Rubinstein M. Kravitz A. V. (2016). Enhanced Gaba Transmission Drives Bradykinesia Following Loss of Dopamine D2 Receptor Signaling. Neuron 90, 824–838. 10.1016/j.neuron.2016.04.040 27196975 PMC4882167

[B31] Levy R. Ashby P. Hutchison W. D. Lang A. E. Lozano A. M. Dostrovsky J. O. (2002). Dependence of Subthalamic Nucleus Oscillations on Movement and Dopamine in Parkinson's Disease. Brain 125, 1196–1209. 10.1093/brain/awf128 12023310

[B32] Lourens M. A. J. Schwab B. C. Nirody J. A. Meijer H. G. E. van Gils S. A. (2015). Exploiting Pallidal Plasticity for Stimulation in Parkinson's Disease. J. Neural Eng. 12, 026005. 10.1088/1741-2560/12/2/026005 25650741

[B33] Lubenov E. V. Siapas A. G. (2008). Decoupling through Synchrony in Neuronal Circuits with Propagation Delays. Neuron 58, 118–131. 10.1016/j.neuron.2008.01.036 18400168

[B34] Madadi Asl M. Vahabie A. H. Valizadeh A. (2019). Dopaminergic Modulation of Synaptic Plasticity, its Role in Neuropsychiatric Disorders, and its Computational Modeling. Basic Clin. Neurosci. 10, 1–12. 10.32598/bcn.9.10.125 31031889 PMC6484184

[B35] Madadi Asl M. Ramezani Akbarabadi S. (2021). Voltage-dependent Plasticity of Spin-Polarized Conductance in Phenyl-Based Single-Molecule Magnetic Tunnel Junctions. PLoS ONE 16, e0257228. 10.1371/journal.pone.0257228 34506579 PMC8432808

[B36] Madadi Asl M. Vahabie A. H. Valizadeh A. Tass P. A. (2022). Spike-timing-dependent Plasticity Mediated by Dopamine and its Role in Parkinson’s Disease Pathophysiology. Front. Netw. Physiology 2, 1–18. 10.3389/fnetp.2022.817524 PMC10013044 36926058

[B37] Madadi Asl M. Valizadeh A. Tass P. A. (2018a). Delay-induced Multistability and Loop Formation in Neuronal Networks with Spike-timing-dependent Plasticity. Sci. Rep. 8, 12068. 10.1038/s41598-018-30565-9 30104713 PMC6089910

[B38] Madadi Asl M. Valizadeh A. Tass P. A. (2017). Dendritic and Axonal Propagation Delays Determine Emergent Structures of Neuronal Networks with Plastic Synapses. Sci. Rep. 7, 39682. 10.1038/srep39682 28045109 PMC5206725

[B39] Madadi Asl M. Valizadeh A. Tass P. A. (2018b). Dendritic and Axonal Propagation Delays May Shape Neuronal Networks with Plastic Synapses. Front. Physiol. 9, 1849. 10.3389/fphys.2018.01849 30618847 PMC6307091

[B40] Madadi Asl M. Valizadeh A. Tass P. A. (2018c). Propagation Delays Determine Neuronal Activity and Synaptic Connectivity Patterns Emerging in Plastic Neuronal Networks. Chaos 28, 106308. 10.1063/1.5037309 30384625

[B41] Magill P. J. Bolam J. P. Bevan M. D. (2001). Dopamine Regulates the Impact of the Cerebral Cortex on the Subthalamic Nucleus-Globus Pallidus Network. Neuroscience 106, 313–330. 10.1016/s0306-4522(01)00281-0 11566503

[B42] Mallet N. Micklem B. R. Henny P. Brown M. T. Williams C. Bolam J. P. (2012). Dichotomous Organization of the External Globus Pallidus. Neuron 74, 1075–1086. 10.1016/j.neuron.2012.04.027 22726837 PMC3407962

[B43] Mallet N. Pogosyan A. Márton L. F. Bolam J. P. Brown P. Magill P. J. (2008a). Parkinsonian Beta Oscillations in the External Globus Pallidus and Their Relationship with Subthalamic Nucleus Activity. J. Neurosci. 28, 14245–14258. 10.1523/jneurosci.4199-08.2008 19109506 PMC4243385

[B44] Mallet N. Pogosyan A. Sharott A. Csicsvari J. Bolam J. P. Brown P. (2008b). Disrupted Dopamine Transmission and the Emergence of Exaggerated Beta Oscillations in Subthalamic Nucleus and Cerebral Cortex. J. Neurosci. 28, 4795–4806. 10.1523/jneurosci.0123-08.2008 18448656 PMC6670450

[B45] Markram H. Lübke J. Frotscher M. Sakmann B. (1997). Regulation of Synaptic Efficacy by Coincidence of Postsynaptic Aps and Epsps. Science 275, 213–215. 10.1126/science.275.5297.213 8985014

[B46] McGregor M. M. Nelson A. B. (2019). Circuit Mechanisms of Parkinson's Disease. Neuron 101, 1042–1056. 10.1016/j.neuron.2019.03.004 30897356

[B47] Miguelez C. Morin S. Martinez A. Goillandeau M. Bezard E. Bioulac B. (2012). Altered Pallido-Pallidal Synaptic Transmission Leads to Aberrant Firing of Globus Pallidus Neurons in a Rat Model of Parkinson's Disease. J. Physiology 590, 5861–5875. 10.1113/jphysiol.2012.241331 PMC3528996 22890706

[B48] Moran R. J. Mallet N. Litvak V. Dolan R. J. Magill P. J. Friston K. J. (2011). Alterations in Brain Connectivity Underlying Beta Oscillations in Parkinsonism. PLoS Comput. Biol. 7, e1002124. 10.1371/journal.pcbi.1002124 21852943 PMC3154892

[B49] Neumann W.-J. Staub-Bartelt F. Horn A. Schanda J. Schneider G.-H. Brown P. (2017). Long Term Correlation of Subthalamic Beta Band Activity with Motor Impairment in Patients with Parkinson's Disease. Clin. Neurophysiol. 128, 2286–2291. 10.1016/j.clinph.2017.08.028 29031219 PMC5779610

[B50] Pawlak V. Kerr J. N. D. (2008). Dopamine Receptor Activation Is Required for Corticostriatal Spike-timing-dependent Plasticity. J. Neurosci. 28, 2435–2446. 10.1523/jneurosci.4402-07.2008 18322089 PMC6671189

[B51] Pfeifer K. J. Kromer J. A. Cook A. J. Hornbeck T. Lim E. A. Mortimer B. J. (2021). Coordinated Reset Vibrotactile Stimulation Induces Sustained Cumulative Benefits in Parkinson’s Disease. Front. Physiology 12, 200. 10.3389/fphys.2021.624317 PMC8055937 33889086

[B52] Plenz D. Kital S. T. (1999). A Basal Ganglia Pacemaker Formed by the Subthalamic Nucleus and External Globus Pallidus. Nature 400, 677–682. 10.1038/23281 10458164

[B53] Popovych O. V. Tass P. A. (2012). Desynchronizing Electrical and Sensory Coordinated Reset Neuromodulation. Front. Hum. Neurosci. 6, 58. 10.3389/fnhum.2012.00058 22454622 PMC3308339

[B54] Schwab B. C. König P. Engel A. K. (2021). Spike-timing-dependent Plasticity Can Account for Connectivity Aftereffects of Dual-Site Transcranial Alternating Current Stimulation. NeuroImage 237, 118179. 10.1016/j.neuroimage.2021.118179 34015486

[B55] Shen W. Flajolet M. Greengard P. Surmeier D. J. (2008). Dichotomous Dopaminergic Control of Striatal Synaptic Plasticity. Science 321, 848–851. 10.1126/science.1160575 18687967 PMC2833421

[B56] Song S. Miller K. D. Abbott L. F. (2000). Competitive Hebbian Learning through Spike-timing-dependent Synaptic Plasticity. Nat. Neurosci. 3, 919–926. 10.1038/78829 10966623

[B57] Syrkin-Nikolau J. Neuville R. O'Day J. Anidi C. Miller Koop M. Martin T. (2018). Coordinated Reset Vibrotactile Stimulation Shows Prolonged Improvement in Parkinson's Disease. Mov. Disord. 33, 179–180. 10.1002/mds.27223 29150859 PMC5836884

[B58] Tass P. A. (2003). A Model of Desynchronizing Deep Brain Stimulation with a Demand-Controlled Coordinated Reset of Neural Subpopulations. Biol. Cybern. 89, 81–88. 10.1007/s00422-003-0425-7 12905037

[B59] Tass P. A. Majtanik M. (2006). Long-term Anti-kindling Effects of Desynchronizing Brain Stimulation: a Theoretical Study. Biol. Cybern. 94, 58–66. 10.1007/s00422-005-0028-6 16284784

[B60] Tass P. A. Qin L. Hauptmann C. Dovero S. Bezard E. Boraud T. (2012). Coordinated Reset Has Sustained Aftereffects in Parkinsonian Monkeys. Ann. Neurol. 72, 816–820. 10.1002/ana.23663 23280797

[B61] Terman D. Rubin J. E. Yew A. C. Wilson C. J. (2002). Activity Patterns in a Model for the Subthalamopallidal Network of the Basal Ganglia. J. Neurosci. 22, 2963–2976. 10.1523/jneurosci.22-07-02963.2002 11923461 PMC6758326

[B62] Vogels T. P. Sprekeler H. Zenke F. Clopath C. Gerstner W. (2011). Inhibitory Plasticity Balances Excitation and Inhibition in Sensory Pathways and Memory Networks. Science 334, 1569–1573. 10.1126/science.1211095 22075724

[B63] Wang J. Nebeck S. Muralidharan A. Johnson M. D. Vitek J. L. Baker K. B. (2016). Coordinated Reset Deep Brain Stimulation of Subthalamic Nucleus Produces Long-Lasting, Dose-dependent Motor Improvements in the 1-Methyl-4-Phenyl-1,2,3,6-Tetrahydropyridine Non-human Primate Model of Parkinsonism. Brain Stimul. 9, 609–617. 10.1016/j.brs.2016.03.014 27151601 PMC10226766

[B64] Woodin M. A. Ganguly K. Poo M. M. (2003). Coincident Pre- and Postsynaptic Activity Modifies GABAergic Synapses by Postsynaptic Changes in Cl- Transporter Activity. Neuron 39, 807–820. 10.1016/s0896-6273(03)00507-5 12948447

